# Efficient metabolic evolution of engineered *Yarrowia lipolytica* for succinic acid production using a glucose-based medium in an in situ fibrous bioreactor under low-pH condition

**DOI:** 10.1186/s13068-018-1233-6

**Published:** 2018-08-30

**Authors:** Chong Li, Shi Gao, Xiaotong Li, Xiaofeng Yang, Carol Sze Ki Lin

**Affiliations:** 1 School of Energy and Environment, City University of Hong Kong, Tat Chee Avenue, Kowloon, Hong Kong; 2 Agricultural Genomic Institute at Shenzhen, Chinese Academy of Agricultural Sciences, Shenzhen, 518120 Guangdong People’s Republic of China; 3 School of Biology and Biological Engineering, South China University of Technology, Guangzhou, 510006 People’s Republic of China

**Keywords:** Metabolic evolution, Succinic acid, *Yarrowia lipolytica*, In-situ fibrous bed bioreactor

## Abstract

**Background:**

Alkali used for pH control during fermentation and acidification for downstream recovery of succinic acid (SA) are the two largest cost contributors for bio-based SA production. To promote the commercialization process of fermentative SA, the development of industrially important microorganisms that can tolerate low pH has emerged as a crucial issue.

**Results:**

In this study, an in situ fibrous bed bioreactor (isFBB) was employed for the metabolic evolution for selection of *Y. lipolytica* strain that can produce SA at low pH using glucose-based medium. An evolved strain named *Y. lipolytica* PSA3.0 that could produce SA with a titer of 19.3 g/L, productivity of 0.52 g/L/h, and yield of 0.29 g/g at pH 3.0 from YPD was achieved. The enzyme activity analysis demonstrated that the pathway from pyruvate to acetate was partially blocked in *Y. lipolytica* PSA3.0 after the evolution, which is beneficial to cell growth and SA production at low pH. When free-cell batch fermentations were performed using the parent and evolved strains separately, the evolved strain PSA3.0 produced 18.4 g/L SA with a yield of 0.23 g/g at pH 3.0. Although these values were lower than that obtained by the parent strain PSA02004 at its optimal pH 6.0, which were 25.2 g/L and 0.31 g/g, respectively, they were 4.8 and 4.6 times higher than that achieved by PSA02004 at pH 3.0. By fed-batch fermentation, the resultant SA titer of 76.8 g/L was obtained, which is the highest value that ever achieved from glucose-based medium at low pH, to date. When using mixed food waste (MFW) hydrolysate as substrate, 18.9 g/L SA was produced with an SA yield of 0.38 g/g, which demonstrates the feasibility of using low-cost glucose-based hydrolysate for SA production by *Y.* *lipolytica* in a low-pH environment.

**Conclusions:**

This study presents an effective and efficient strategy for the evolution of *Y. lipolytica* for SA production under low-pH condition for the first time. The isFBB was demonstrated to improve the metabolic evolution efficiency of *Y. lipolytica* to the acidic condition. Moreover, the acetate accumulation was found to be the major reason for the inhibition of SA production at low pH by *Y. lipolytica*, which suggested the direction for further metabolic modification of the strain for improved SA production. Furthermore, the evolved strain *Y. lipolytica* PSA3.0 was demonstrated to utilize glucose-rich hydrolysate from MFW for fermentative SA production at low pH. Similarly, *Y.* *lipolytica* PSA3.0 is expected to utilize the glucose-rich hydrolysate generated from other carbohydrate-rich waste streams for SA production. This study paves the way for the commercialization of bio-based SA and contributes to the sustainable development of a green economy.

**Electronic supplementary material:**

The online version of this article (10.1186/s13068-018-1233-6) contains supplementary material, which is available to authorized users.

## Background

Green technology is becoming a driving force in most industrial sectors because of the current demand to decrease the pollution caused by chemical processing, and the future needs to replace the environmental unsustainable hydrocarbon economy with a renewable and environmentally sound carbohydrate economy. As a building block compound which is currently produced by petroleum-based processes, bio-based succinic acid has attracted increasing interest due to its industrial potential for cleaner production and extensive applications in the food and pharmaceutical industries [1], which leads to rapid expansion of market size in recent years. By 2015, the production of bio-based products was around 150 kilotons, and it is predicted to reach 710.0 kilotons by 2020, growing at a compound annual growth rate of 45.6% [2]. Despite the great demand for SA, the current status of microbiological production is still not economically competitive as compared to the traditional petro-chemical synthesis.

Downstream processing is the most expensive step which affects the final cost of bio-based SA and accounts for 60–70% of the overall production cost. Among all unit operation within the process, the acidification of succinate salt from fermentation broth for separation of succinic acid crystals is one of the most costly steps [3]. To date, the bacterial strains such as *Actinobacillus succinogenes* and *Escherichia coli* are considered to be feasible succinate producers. However, they are still unable to grow effectively in a low-pH environment [3, 4], as the excess requirement of energy production for effective retention of intracellular pH and transmembrane gradient of acid is still a question for anaerobic processes due to insufficient ATP generation [5]. In contrast, eukaryotic microorganisms which are known to maintain intracellular pH efficiently could be preferable for such conditions [5]. As an unconventional yeast that is regarded as Generally Recognized As Safe (GRAS) [6], *Yarrowia lipolytica* has been demonstrated to be suitable for SA fermentation with high yield and productivity under neutral condition [7–14]. More importantly, engineered *Y. lipolytica* that can produce SA at low pH has been successfully constructed [5, 15], which demonstrated the feasibility of using *Y. lipolytica* for SA production under low-pH condition. This also led to subsequent reduction in production cost which would bridge the gap between research and commercialization of bio-based SA production.

Nevertheless, owing to the inactivation of the succinate dehydrogenase subunit (SDH), the genetically modified *Y. lipolytica* reported in the literature mainly uses glycerol as the carbon source for SA production [5, 7, 8, 10], and it was regarded as unable to grow well in glucose-based medium which results from the loss of the FAD/FADH_2_ recycling pathway [9, 16, 17]. As a feedstock that was commonly used for fermentative SA production [18–21], glucose is more accessible and could even be obtained from waste streams [22–25]. Very recently, Bondarenko et al. reported a glucose-consuming *Y. lipolytica* VKPM Y3753 that can produce SA at pH 3.65 was achieved using an engineered *Y. lipolytica* VKPM Y3314 which was genetically modified by multistage mutagenesis [26]. Although with lower SA production and higher fermentation pH than that reported by Cui et al. [15] and Yuzbashev et al. [5], the study demonstrated the feasibility of using engineered *Y. lipolytica* for SA production using glucose-based medium [26]. Recently, a *Y. lipolytica* strain PSA02004 that is able to grow and produce SA in glucose-based medium under neutral conditions has been developed in our group [17]. Therefore, it would be greatly beneficial if fermentative SA production using glucose-based medium by *Y. lipolytica* can be achieved in low-pH environment.

Metabolic evolution is an approach to strain improvement for metabolic engineering applications that utilizes a microorganism’s natural ability to grow and evolve [27]. Different from the genetic modification applied in Yuzbashev’s study [5], the evolutionary phenotype changes and selection via metabolic evolution based on cell growth behavior was identified as a suitable approach for generating an improved strain with the desired metabolic pathways [27–30]. As an efficient way to gain advantageous mutations through positive selection, it has been successfully used to improve the production of many fermentation products [31–35]. In our previous study, it was successfully used for restoration of glucose metabolism in *Y. lipolytica* for SA production [17]. On the other hand, as a newly developed fermentation mode which consists of a normal 2.5-L fermenter embedded with immobilized matrix, the in situ fibrous bed (isFBB) fermentation has been demonstrated not only to enhance product production and prevent contamination, but also improve the efficiency of metabolic evolution [7, 8, 17, 36–38]. In this case, it is expected that the combination of isFBB fermentation and metabolic evolution would provide new avenues for screening and selection of *Y. lipolytica* with the desired phenotypic trait and characteristic.

The objective of this study was to obtain an evolved *Y. lipolytica* strain that can produce SA from glucose-based substrate at low pH via metabolic evolution. First, metabolic evolution of the parent strain *Y. lipolytica* PSA02004 was carried out via repeated isFBB fermentation with YPD as the cultivation medium for strain selection with a unique character for producing SA steadily under low-pH conditions. Then, the evolved strain PSA3.0 was cultivated in YPD with various initial glucose concentrations. Afterwards, fed-batch fermentation was employed to achieve a high SA production by the evolved PSA3.0 at low pH. Finally, the feasibility of using glucose-rich hydrolysate from food waste for SA production by PSA3.0 at low pH was investigated. Upon successful implementation of metabolic evolution, an improved strain that could produce SA from glucose-based medium at low pH was screened and selected.

## Results and discussion

### Metabolic evolution of *Y. lipolytica* for SA production at low pH

#### Metabolic evolution of *Y. lipolytica* by repeated batch fermentation

Repeated batch fermentation in isFBB was applied to perform metabolic evolution of *Y. lipolytica* to obtain an evolved strain that can produce SA at low pH using glucose-based medium. *Y. lipolytica* PSA02004 that can consume glucose for SA production under the neutral condition (pH 6.0) was used as the parent strain in this study [17]. The Additional file 1: Table S1 shows the number of repeated batch fermentation, their corresponding pH, and the name of evolved strains.

**Fig. 1:**
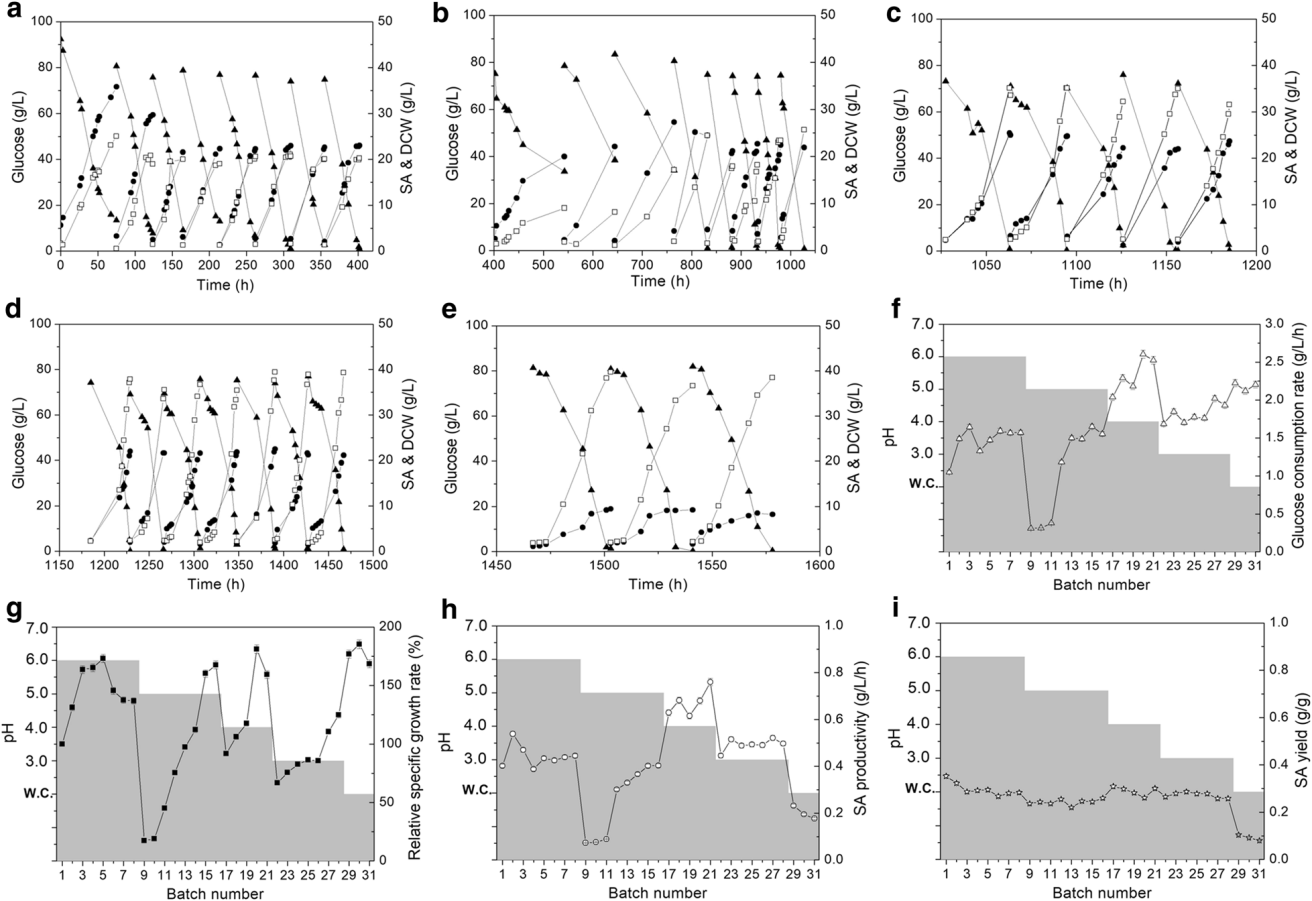
Metabolic evolution of *Y. lipolytica* PSA02004 for SA production at low pH. **a** Cell growth and SA production at pH 6.0. **b** Cell growth and SA production at pH 5.0. **c** Cell growth and SA production at pH 4.0. **d** Cell growth and SA production at pH 3.0. **e** Cell growth and SA production at pH without control. W.C refers to without pH control. **f** Glucose consumption rate at different pH. **g** Relative specific growth rate at different pH, in which the specific growth rate of the first generation was set as 100%. Relative specific growth rate (%) = (specific growth rate in any generation)/(specific growth rate in the first generation) × 100. **h**, **i** SA productivity and SA yield at different pH (filled triangles represent glucose concentration; filled circles represent SA concentration; open squares represent DCW; shaded areas represent the corresponding pH; open triangles represent glucose consumption rate; filled square represents the relative specific growth rate; open circles represent SA productivity; open stars represent SA yield)

First, the *Y. lipolytica* PSA02004 was cultivated in repeated batch fermentation at its optimal pH 6.0 for eight batches. As shown in Fig. 1a, the cell growth and SA production of PSA02004 varied in the first three batches. Afterwards, cell growth and SA production of PSA02004 became stable during the 4th–8th batches. As shown in Fig. 1a, glucose was completely consumed in about 45 h, and their corresponding glucose consumption rates were around 1.5 g/L/h (Fig. 1f). By setting the specific growth rate of the first batch (0.18 h^−1^) as 100% (Fig. 1g), the relative specific growth rates fluctuated between 125 and 175%, and the average DCW around 20 g/L were obtained (Fig. 1a) in these batches. On the other hand, the SA production in titer, productivity, and yield were stabilized at around 20.0 g/L, 0.43 g/L/h, and 0.28 g/g, respectively. Since that stable cell growth and SA production were obtained at this pH 6.0, pH of the fermentations was decreased to 5.0 from the 9th batch. As shown in Table 1, high acetate concentration (15.7 g/L) was obtained at pH 6.0.

**Table 1:** Summary of SA fermentation at different pH via metabolic evolution

pH	Batch number	Glucose consumption rate (g/L/h)	Relative specific growth rate^a^ (100%)	SA titer (g/L)	SA productivity (g/L/h)	SA yield (g/g)	Pyruvate (g/L)	Acetate (g/L)
6	1–8	1.46 ± 0.18	144 ± 22	20.0 ± 0.7	0.44 ± 0.04	0.30 ± 0.03	2.5 ± 0.2	15.7 ± 1.6
5	9–16	1.05 ± 0.57	87 ± 56	18.7 ± 1.2	0.36 ± 0.14	0.24 ± 0.01	6.7 ± 0.2	3.2 ± 0.6
4	17–21	2.33 ± 0.21	131 ± 34	21.1 ± 1.2	0.67 ± 0.05	0.29 ± 0.02	5.9 ± 0.2	N.D
3	22–28	1.82 ± 0.11	90 ± 19	19.7 ± 0.5	0.49 ± 0.02	0.27 ± 0.01	1.0 ± 0	N.D
Without control (i.e. 2.1–2.5)	29–31	2.18 ± 0.05	177 ± 7	7.4 ± 0.7	0.20 ± 0.02	0.09 ± 0.01	1.0 ± 0.1	N.D

^a^The relative specific growth rate was calculated by setting the specific growth rate of the first batch (0.18 h^−1^) as 100%

When pH was decreased to 5.0 from the 9th batch, inadaptability of the strain was observed. To be more specific, the fermentation time increased from about 45 h at pH 6.0 to over 140 h at pH 5.0 in the 9th batch, in which around half of the glucose (31.5 g/L) in broth was still remained unconsumed (Fig. 1b). Correspondingly, SA productivity decreased by 83.3% to 0.07 g/L/h as compared to the 8th batch at pH 6.0 (Fig. 1h). In addition, a very low relative specific growth rate of 17.5% was recorded in this batch (Fig. 1g). The negative influence of the acidic condition to the strain lasted for three batches (Fig. 1b, g–i) before the cell growth and SA production recovered (Fig. 1b). It should be pointed out that no obvious decline in SA yield was recorded when the pH decreased to pH 5.0 in the 9th batch (Fig. 1i), this indicated that the strain was not damaged. From the 12th batch, cell growth and SA production were fully recovered (i.e., glucose was completely depleted in around 45 h again, as shown in Fig. 1b), while SA production became stable at around 18.0–20.0 g/L in titer and 0.35–0.40 g/L/h in productivity (Fig. 1h). Moreover, the relative specific growth rate increased to 100% in the 14th batch and even higher in the last two batches (i.e., 15th and 16th batches) at pH 5.0 (Fig. 1g). These indicated that the stable cell growth and SA production at pH 5.0 were obtained, and the evolved strain was named as ‘PSA5.0’ (Additional file 1: Table S1).

**Fig. 2:**
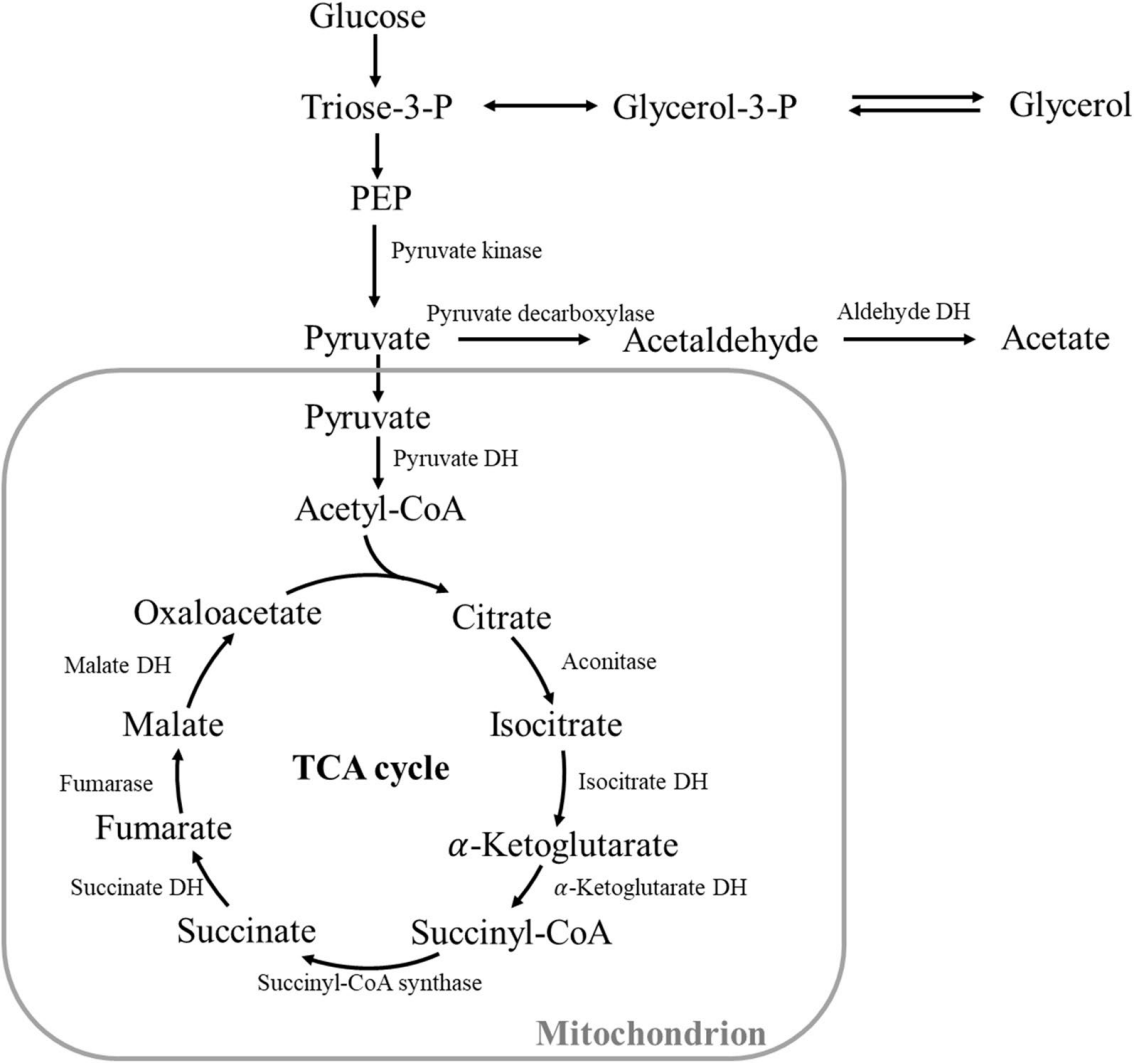
Metabolic pathways for succinate production in *Y. lipolytica*

On the other hand, when the fermentation pH was decreased from 6.0 to 5.0, acetate production dropped sharply to 3.2 g/L, while pyruvate production increased from 2.5 to 6.7 g/L (Table 1). According to the metabolic pathways of succinate production in *Y. lipolytica* [15, 17], it may be anticipated that the conversion from pyruvate to acetate was partially blocked in PSA5.0 due to reduced enzyme activities by lowering the environmental pH condition (Fig. 2). It should be pointed out that the relatively high SA conversion rates in the 9th–11th batches should be partially contributed by water evaporation during the extended fermentation period at pH 5.

After the progressive selection of the evolved PSA5.0 strain with phenotype improvement for stable SA production at pH 5.0, the fermentation pH was decreased stepwise to 4.0 in the 17th–21st batches. Figure 1c depicts no significant inhibition on cell growth or SA production was observed. In contrast, a significant increase in cell growth (31.5–35.1 g/L DCW, Fig. 1a–c), glucose consumption rate (2.18–2.60 g/L/h, Fig. 1f), and SA productivity (0.62–0.76 g/L/h, Fig. 1h) were observed as compared to that obtained at pH 5.0. This evolved strain that could grow and produce SA stably at pH 4.0 was designated as ‘PSA4.0’ (Additional file 1: Table S1). Another interesting phenomenon observed in Table 1 was no acetate was detected, but the pyruvate concentration remained at a high level of 5.9 g/L at pH 4, which suggested that the pathway from pyruvate to acetate remained blocked. This may indicate that the inhibition of microbial growth and SA production under low-pH condition was attributed to the presence of acetate (i.e., carboxyl group) instead of the acidic condition (i.e., hydronium ion) [5, 15].

After generating the evolved strain PSA4.0 via incremental reduction in pH to progressively improve the pH tolerance of *Y. lipolytica* strain, the fermentation pH was further declined to 3.0 in the 22nd–28th batches. As shown in Fig. 1d, h, i, the decrease of pH from 4.0 to 3.0 resulted in no significant change to SA production, and the corresponding SA production in titer, productivity, and yield were around 19.3 g/L, 0.52 g/L/h, and 0.29 g/g, respectively. However, a significant increase in biomass accumulation was resulted, which might be the coupled response of this strain to the acidic condition. Meanwhile, pyruvate production decreased to a level below 1.0 g/L at pH 3.0. These indicate that more carbon source was distributed to biomass accumulation instead of going through the TCA cycle for SA production. The evolved strain selected at pH 3.0 was titled ‘PSA3.0’ (Additional file 1: Table S1).

Finally, the fermentation pH was decreased to the range of 2.1–2.5 (without pH control) in the 29th–31st batches. As shown in Fig. 1e, SA titer decreased by 60.2% to around 7.4 g/L as compared to that obtained at pH 3.0. Accordingly, SA productivity decreased by over 50% to around 0.20 g/L/h (Fig. 1h). As shown in Fig. 1I, SA yield decreased to below 0.10 g/g in this pH range. In contrast, DCW and the relative specific growth rate remained at high levels (Fig. 1e, g), which demonstrated that most substrate was used for biomass formation instead of SA production in this pH range. This result agrees well with the former studies in bacteria, in which the metabolic evolution is beneficial to ATP generation and thus contributes to microbial growth [28, 29]. However, it is controversial to the study which reported that cells spend more energy on catabolism (i.e., to favor succinate production) at low pH [5]. Due to poor SA production which was resulted at this pH range, metabolic evolution was stopped at this stage and the evolved strain was titled ‘PSA 2.5’ in Additional file 1: Table S1.

After the metabolic evolution, the evolved strain PSA 3.0 that could steadily grow and produce SA at pH 3.0 was selected for the following experiments for the balance between cell growth and SA production.

#### Evaluation of the metabolic evolved strain

To evaluate the evolved strain PSA3.0, comparisons in cell growth and SA production between PSA3.0 and the parent strain PSA02004 were conducted by free-cell batch fermentation at pH 3.0 and pH 6.0 (i.e., at the optimal pH of 6.0 for PSA02004 and pH 3.0 for both strains). As shown in Table 2, the evolved strain PSA3.0 grew well at pH 3.0. After 34.5 h, glucose was completely depleted by PSA3.0 and the DCW was 1.5 times higher than that obtained by PSA02004, even at its optimal pH of 6.0. At pH 3.0, poor cell growth of PSA02004 was observed and the DCW only reached at 1.5 g/L after 50 h, which was much lower than that achieved by PSA3.0 at pH 3.0, and thus, it indicates the improved pH tolerance of the evolved strain PSA3.0 in cell growth at low-pH environment.

**Table 2:** Cell growth and SA production of *Y. lipolytica* PSA02004 and PSA3.0 at different pH

Strain	pH	Fermentation time (h)	Glucose consumption (g/L)	SA titer (g/L)	SA yield (g/g)	SA productivity (g/L/h)	DCW (g/L)	Acetate (g/L)
PSA02004	6	50 ± 0.5	81.5 ± 1.5	25.2 ± 0.5	0.31 ± 0.01	0.50 ± 0.02	24.5 ± 1.5	11.4 ± 1.0
	3	50 ± 0.5	8.6 ± 0.5	3.8 ± 0.3	0.05 ± 0.01	0.08 ± 0.01	1.5 ± 0.5	4.4 ± 0.5
PSA3.0	3	34.5 ± 1.0	80.9 ± 1.0	18.4 ± 0.5	0.23 ± 0.01	0.53 ± 0.03	37.7 ± 2.5	0

When comparing SA production of the strains, 18.4 g/L SA with a yield of 0.23 g/g was produced by PSA3.0 at pH 3.0, which were 27.0% and 25.8% lower than that achieved by PSA02004 at its optimal pH of 6.0, respectively. Nevertheless, the SA production by PSA3.0 at pH 3.0 was 4.8, 4.6, and 6.6 times of those obtained by PSA02004 at pH 3.0 in terms of SA titer, yield, and productivity, respectively. Moreover, there was essentially no acetate which was produced by PSA3.0, the downstream process for SA recovery from fermentation broth of PSA3.0 would be simpler than that using fermentation broth of PSA02004 [15, 39]. The results demonstrate the stronger ability of PSA3.0 than the parent strain PSA02004 in SA production at low pH, and it also indicates the success of metabolic evolution.

#### Comparison of enzyme activities in the initial and evolved strains

To assure the reason for the disappearance of acetate production in PSA3.0, the activities of two enzymes including pyruvate decarboxylase (PDC) and aldehyde dehydrogenase (ALDH), those are responsible for the conversion of pyruvate to acetate in *Y.* *lipolytica* were determined and analyzed in the parent and evolved strains, respectively [15]. As shown in Table 3, although PDC activity was present in both strains, the value was 39.2% higher in PSA02004 than that in PSA3.0, which indicated that the activity of PDC may be reduced with the decrease of pH during metabolic evolution. On the other hand, no ALDH activity was detected from PSA3.0, suggesting that its activity also declined with the decrease of pH during the evolution.

**Table 3:** Enzyme activity measurement of *Y. lipolytica* PSA02004 and PSA3.0

Strain	pH	PDC (U/g DCW)	ALDH (U/g DCW)
PSA02004	6.0	427.9 ± 76.9	1.2 ± 0.1
PSA3.0	3.0	307.5 ± 39.7	N.D

*PDC* pyruvate decarboxylase, *ALDH* aldehyde dehydrogenase, *N.D* not detected

According to the acetate and pyruvate production summarized in Table 1, it can be inferred that when the pH decreased from 6.0 to 5.0, inadaptability appeared in the strain, and it reorganized the oxidative metabolism to first meet the anabolic requisition for cell growth [40]. Therefore, the activities of PDC and ALDH declined (Table 3), and the formation of acetate decreased sharply from 15.7 to 3.2 g/L. The increased accumulation of pyruvate may result from the impairment of the pathway to acetate. Further decreasing the pH to 4.0 resulted in disappearance of acetate. When the pH decreased to 3.0 (i.e., under an adverse condition), the strain distributed more resource to the anabolic constituents to support cell growth, so the concentrations of metabolites including succinate and pyruvate were even lower than that at pH 6.0, while the DCW doubled.

### Effect of the initial glucose concentration on *Y. lipolytica* PSA3.0

It was reported that a significant amount of byproducts including mannitol, erythritol, and arabitol would be produced from high concentrations of glycerol at low pH by *Y. lipolytica* [15]. As a result, the effect of the initial glucose concentrations on cell growth and SA production of *Y. lipolytica* PSA3.0 was investigated.

According to Table 4, SA titer increased from 6.9 to 18.9 g/L with the rise of the initial glucose concentration from 25 to 75 g/L. Similarly, SA productivity doubled and reached the highest at 0.55 g/L/h when the initial glucose concentration was 75 g/L. Continuously increasing the glucose concentration to 150 g/L led to the decline of both SA titer and productivity to only 13.9 g/L and 0.26 g/L/h, respectively. In contrast, with the rise of glucose concentration from 25 to 150 g/L, SA yield decreased constantly from 0.3 g/g to 0.09 g/g, while DCW increased from 19.9 to 49.5 g/L at the same time. This indicates that although a higher glucose concentration is beneficial to the accumulation of cell mass, a glucose concentration range of 50–75 g/L is preferred for the balance between microbial growth and SA production.

**Table 4:** Effect of initial glucose concentrations on cell growth and SA production of *Y.* *lipolytica* PSA3.0

Initial glucose (g/L)	Glucose consumption (g/L)	Fermentation time (h)	SA titer (g/L)	SA yield (g/g)	SA productivity (g/L/h)	DCW (g/L)	Final pH
25.0 ± 2.5	25.0 ± 2.5	26.0 ± 0.5	6.9 ± 0.5	0.30 ± 0.01	0.27 ± 0.01	19.9 ± 1.5	3.9
50.0 ± 1.5	50.0 ± 1.5	29.0 ± 0.5	12.6 ± 0.3	0.26 ± 0.01	0.43 ± 0.01	33.8 ± 1.5	3.3
75.0 ± 3.1	75.0 ± 3.1	34.5 ± 1.0	18.9 ± 0.5	0.23 ± 0.01	0.55 ± 0.03	37.7 ± 2.5	3.0
100.0 ± 1.5	100.0 ± 1.5	44.5 ± 1.5	15.9 ± 0.2	0.16 ± 0.01	0.36 ± 0.02	48.1 ± 2.5	3.0
150.0 ± 2.5	129.2 ± 1.5	54.0 ± 1.5	13.9 ± 0.6	0.09 ± 0.00	0.26 ± 0.01	49.5 ± 3.0	3.0

On the other hand, apart from pyruvate and acetate that were produced by *Y. lipolytica* PSA02004 at pH 6.0, no additional by-product was detected in SA fermentation by *Y.* *lipolytica* PSA3.0.

### Fed-batch fermentation at low pH for high SA production

Currently, the highest fermentative SA production in titer, either from glucose [41] or glycerol [7], was obtained by the fed-batch fermentation strategy. To achieve a high SA titer at low pH by *Y. lipolytica* PSA3.0, fed-batch fermentation was employed.

**Fig. 3:**
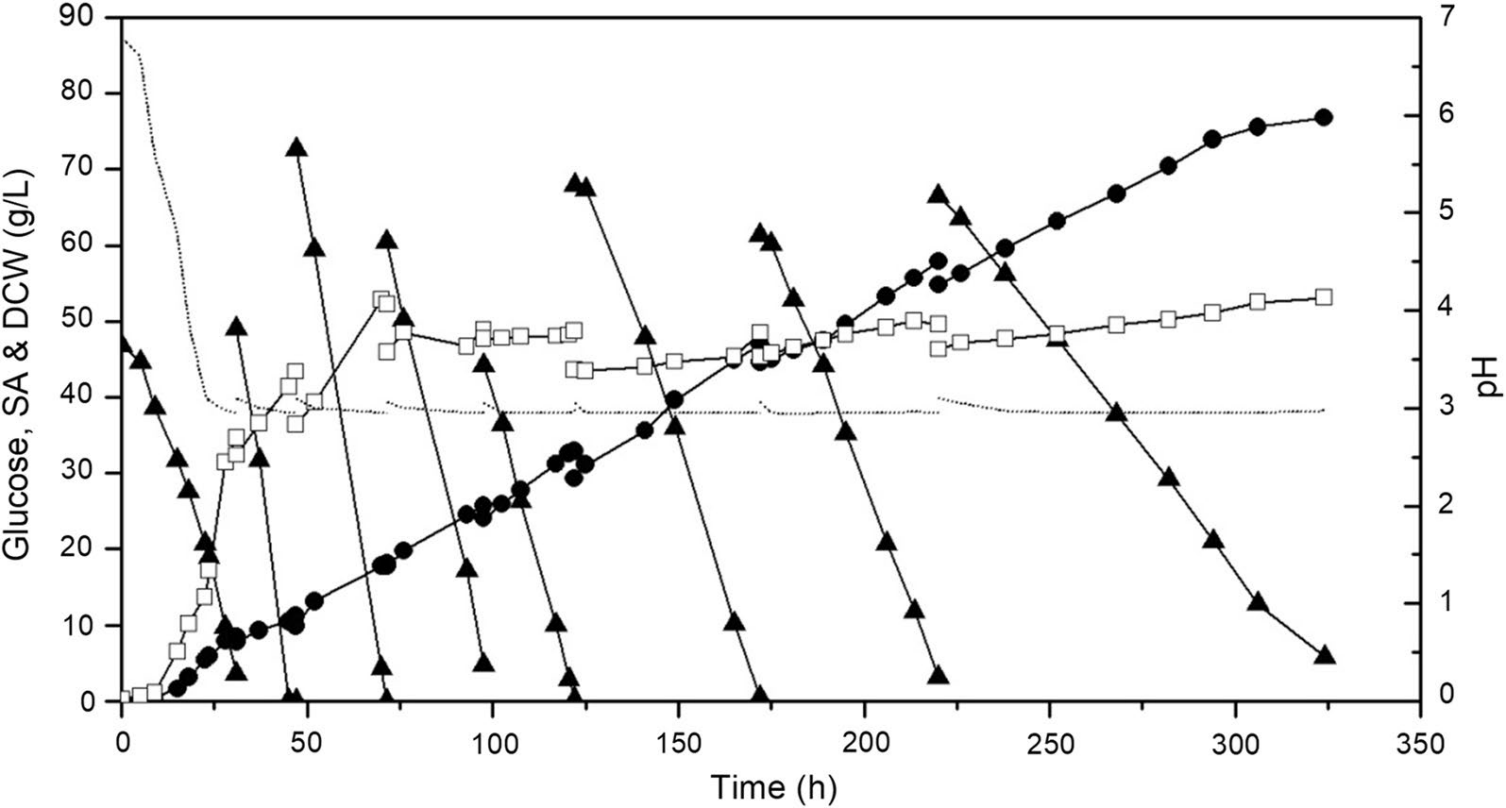
Fed-batch fermentation for SA production by evolved strain PSA 3.0 at low pH. (dotted line represents pH value; filled triangles represent glucose concentration; filled circles represent SA concentration; open squares represent DCW)

As shown in Fig. 3, the pH of the fermentation broth decreased rapidly from 6.8 to the set point of 3.0 before the depletion of glucose in 28 h, and remained at this value till the end of fermentation. With the decrease of pH, DCW increased exponentially from 0.2 to 31.4 g/L, and reached 50 g/L after two feedings of glucose in 70 h. Afterwards, the DCW remained in the range of 45.3–53.1 g/L till the end of fermentation.

SA titer increased gradually with the feeding of glucose. After seven times of feeding, a SA titer of 76.8 g/L resulted in 324 h. This is the highest fermentative SA was titer achieved using glucose-based medium by *Y. lipolytica* at low pH ever reported to date. However, owing to the relatively long fermentation time, the average SA productivity was relatively low (i.e., 0.24 g/L/h). Moreover, resulting from the shift of the metabolic ratio to anabolic requisition for biomass accumulation, a relatively low SA yield of 0.20 g/g was obtained [28, 29, 40]. Nevertheless, the study demonstrated the successful implementation of metabolic evolution of *Y. lipolytica* in the fermentative SA production using glucose-based medium at low pH.

### SA production from glucose-rich hydrolysate at low pH

The high cost of raw materials is one of the major obstacles to the commercialization of bio-SA [9, 23, 42]. In this study, the feasibility of using MFW hydrolysate as substrate for SA production by *Y. lipolytica* at low pH was investigated.

As shown in Table 5, the hydrolysate contains a relatively high initial concentration of glucose of 100.8 g/L. Moreover, free amino nitrogen (FAN) concentration in hydrolysate was 1.7 g/L. The high glucose content combined with the existence of FAN indicated that the hydrolysate would be an ideal cultivation medium and nutrient source in the fermentative production of SA [9]. Apart from glucose, other free sugars such as fructose (1.5 g/L) were also obtained from the hydrolysate, and it might be also used as the carbon source by *Y. lipolytica* in other studies [43, 44].

**Table 5:** Composition of the mixed food waste hydrolysate

Composition	Content
Glucose	100.8 ± 5.0 (g/L)
Fructose	1.5 ± 0.2 (g/L)
FAN	1700 ± 100 (mg/L)
pH	3.9

**Fig. 4:**
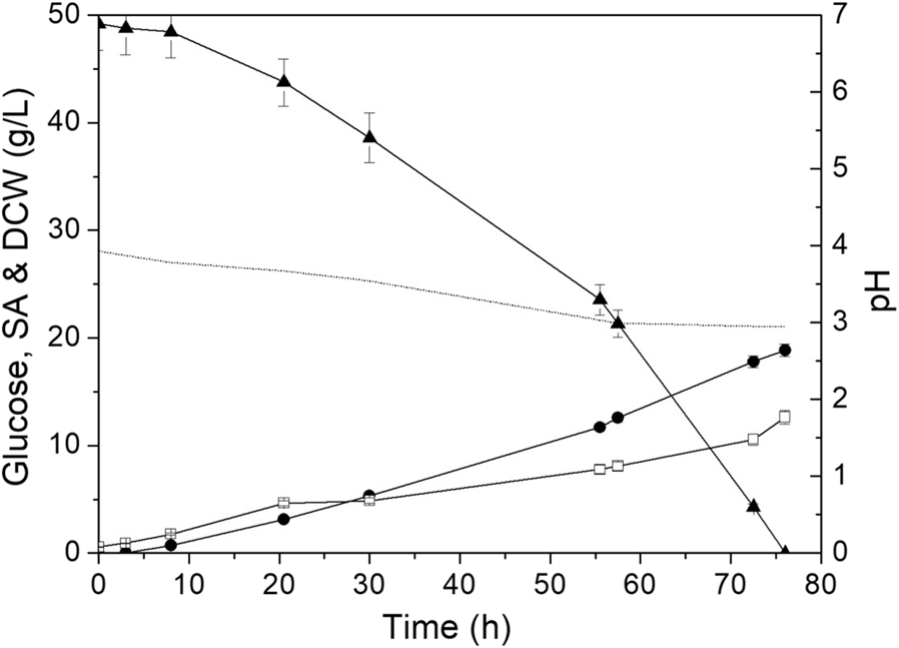
Cell growth and SA production of evolved PSA 3.0 under low-pH condition with mixed food waste hydrolysate as feedstock (dotted line represents pH value; filled triangles represent glucose concentration; filled circles represent SA concentration; open squares represent DCW)

The results for fermentative SA production using the hydrolysate as feedstock by *Y. lipolytica* PSA3.0 at low pH are depicted in Fig. 4. Glucose in the hydrolysate was completely depleted in 76 h, and SA was produced simultaneously with a titer of 18.9 g/L and a yield of 0.38 g/g. This value is similar to that obtained by *Y. lipolytica* PGC01003 using crude glycerol as the carbon source [10], but 46.2% higher than that by *Y. lipolytica* PSA3.0 from the defined medium at pH 3.0 (Table 4). Moreover, when considering the higher resultant DCW (33.8 g/L) of fermentation broth from defined medium at pH 3.0 (Table 4) than that resulting from hydrolysate (i.e., DCW = 12.3 g/L), this indicated that the utilization of hydrolysate as nutrient source would favor SA production instead of cell growth by *Y. lipolytica* PSA3.0. Based on the fermentation results from hydrolysate, it is inferred that *Y. lipolytica* PSA3.0 has a good substrate adaptability for SA production at low pH. It is anticipated that the glucose-rich hydrolysate from other carbohydrate-rich waste streams [45–47] may also be suitable in the fermentative SA production by *Y.* *lipolytica* PSA3.0 at low pH.

## Conclusions

This is the first investigation using metabolic evolution of *Y. lipolytica* in microbial biosynthesis of SA at low pH. The isFBB was demonstrated to improve the metabolic evolution efficiency of *Y. lipolytica*. The evolved strain PSA3.0 was able to produce 19.3 g/L SA with a productivity of 0.52 g/L/h and a yield of 0.29 g/g from glucose at pH 3.0. Moreover, acetate accumulation was found to be the major reason for inhibition of *Y. lipolytica* in cell growth and SA production at low pH, which suggested the direction for further metabolic modification of the strain for improved SA production. Furthermore, the evolved strain *Y. lipolytica* PSA3.0 was demonstrated to utilize glucose-rich hydrolysate from MFW for fermentative SA production at low pH, which indicated the extensive substrate adaptability of the strain. In summary, this study paves the way for the commercialization of bio-based SA, which would contribute to the sustainable development of a green and circular bio-economy.

## Materials and methods

### Strains and medium

The *Y. lipolytica* PSA02004 used as the starting parent strain in this study was obtained in our former study [17]. The seed culture was prepared by incubating the strain from a cryopreservation vial in a 250-mL shake flask containing 50 mL of YPD medium that was comprised of (w/v) 1% yeast extract, 2% tryptone, and 3% glucose at 28 °C and 250 rpm. The fermentation media used in this study were mainly YPD with various initial concentrations of glucose (25–150 g/L) and pH (2.5–6.0). Carbon and nitrogen sources were prepared separately and sterilized at 121 °C for 15 min.

Chemicals were mainly purchased from Oxoid (Basingstoke, UK) or Sigma-Aldrich (MO, USA). The enzymes used in this study were kindly provided by Novozymes^®^ (China). Enzyme assay kits were bought from Beijing Solarbio Science & Technology Co., Ltd. (China). Mixed food waste (MFW) consisting of rice, noodles, meat, and vegetables were collected from Asia Pacific Catering at Hong Kong Science Park.

### Preparation of food waste hydrolysate

Mixed food waste was blended immediately after collection and stored at 4 °C until use. Enzymatic hydrolysis of the MFW and composition determination of the hydrolysate were conducted based on the methods described in our previous study [17, 48]. The MFW was hydrolyzed at a solid-to-liquid ratio of 30% (w/v) in a 2.5-L bioreactor (New Brunswick Scientific, USA) at 50 °C. Enzymes including glucoamylase (1%, v/w), cellulase (1%, v/w), and protease (1%, v/w) were added to the bioreactor when the temperature reached 50 °C. After 24 h when the hydrolysis was completed, hydrolysate samples were taken for composition analysis.

The hydrolysate was sterilized by membrane filtration (0.22 μm, Sartorius, Germany) and stored at − 20 °C until use. The hydrolysate was diluted to obtain glucose at a suitable concentration and supplemented with corn steep liquor (2%, w/v) when necessary [17].

### Construction of an in situ fibrous bed bioreactor

The in situ fibrous bed bioreactor was constructed in a 2.5-L bench-top fermenter (Sartorius Biostat B, Germany). After the pretreatment steps of washing, sterilization by steam, drying, crushing, and sieving, 20 g sugarcane bagasse with a suitable size range of 1.0–1.5 mm was put into silk stockings and stitched onto a piece of stainless steel wire mesh. The mesh was then fixed to the baffle of the fermenter to complete the construction [8].

### Metabolic evolution of *Y. lipolytica* PSA02004 for SA production at low pH by repeated isFBB fermentation

isFBB fermentation is a two-stage process, which consists of immobilization and fermentation stages [8]. When the immobilization stage finished, the fermentation broth was replaced by fresh YPD medium. Once the glucose is nearly depleted (< 5 g/L), the fermentation broth was replaced by fresh YPD medium. The process was repeated to start the repeated isFBB fermentation and metabolic evolution.

For the first several batches, the pH of the YPD for replacement was adjusted to 6.0 in advance. The repeated fermentation was carried out at pH 6.0 several times until stable cell growth and SA production were achieved. Then, the pH of the YPD for replacement was adjusted to 5.0 and repeated fermentation was performed at pH 5.0 until stable cell growth and SA production again. Afterwards, the pH of the YPD for replacement was adjusted to 4.0 or 3.0 for the repeated isFBB fermentation. The repeated fermentation without pH control followed with the fermentations at pH 3.0, and the pH of the YPD for replacement was also adjusted to 3.0. However, the pH was not controlled during the fermentation, so the final pH eventually decreased to the range of 2.1–2.5.

Stable cell growth and SA production refer to the DCW and resultant SA of the last several repeated batches at any specific pH (e.g., pH 6.0 or 5.0) were similar. Glucose concentrations in YPD for replacement were around 80 g/L, and the pH of the fermentation was automatically controlled by adding 5-M sodium hydroxide when necessary.

### Enzyme activity assay

The parent strain *Y. lipolytica* PSA02004 was cultured in a 250-mL shake flask at pH 6.0, and the evolved strain *Y. lipolytica* PSA3.0 was grown at pH 3.0. When they reached the stationary phase, about 10 mL of the culture broth for two strains were harvested and separately suspended in 1-mL extraction buffer consisting of 50-mM Tris–HCl and 20% glycerol at pH 8.0. The cells were broken using 0.4–0.6-mm glass beads (Sigma-Aldrich, St. Louis, MO) at low temperature for 10 min prior to centrifugation. The supernatant was obtained and put on ice for further analysis [15].

The activities of pyruvate decarboxylase (PDC) and aldehyde dehydrogenase (ALDH) were determined by Assay Kits (Cat Number: BC1070 and BC0750, respectively; Solarbio Science & Technology Co., Ltd., Beijing, China). The principle of these methods was based on spectrophotometry, and detailed description is provided in the specifications. In general, the crude enzyme solutions were obtained and they were mixed with special reagents that provided in the kits, and then, OD_340_ was measured per minute. The difference in OD_340_ at various time intervals was used for enzyme activities calculation based on the corresponding equations provided in the specifications. The same procedures were conducted for the control group, except that the crude enzyme solution was replaced by DI water.

### Free-cell batch fermentation

To evaluate the results of the metabolic evolution, free-cell batch fermentation was carried out for SA production using YPD with an initial glucose concentration of 80 g/L at pH 3.0 by the evolved strain *Y. lipolytica* PSA3.0. Fermentations with the initial strain of *Y.* *lipolytica* PSA02004 at pH 3.0 and 6.0 were set as the control. The free-cell batch fermentations were performed in 2.5-L bench-top fermenters with a working volume of 1.0 L at 28 °C, with an agitation speed of 600 rpm and aeration rate of 2 L/min.

To investigate the influence of the glucose concentration on cell growth and SA production of the evolved strain PSA3.0, fermentations were carried out with various glucose concentrations from 25 to 150 g/L. The initial pH of the YPD media was between 6.0 and 7.0 after autoclaving, and decreased naturally until pH 3.0 where it was controlled.

To test the feasibility of using cheap substrates for SA production at low pH, hydrolysate containing an initial glucose concentration of 50 g/L from mixed food waste was used as feedstock for fermentative SA production by *Y. lipolytica* PSA3.0. Corn steep liquor (2%, w/v) was supplemented as the nitrogen source.

### Fed-batch fermentation for SA production

Fed-batch fermentation was carried out in a 2.5-L bench-top bioreactor at 28 °C, with an agitation speed of 600 rpm and aeration rate of 2 L/min. The initial glucose concentration of YPD medium was 50 g/L, and about 80–100 mL of 500 g/L glucose was fed to supplement the carbon source when the glucose concentration was below 10 g/L.

### Analytical methods

Microbial growth was determined by OD_600_ and DCW [8]. The specific growth rate (*μ*) was calculated by the following equation:

1 $$ \mu = \frac{1}{X} \times \frac{{{\text{d}}X}}{{{\text{d}}t}}, $$

where *X* is DCW and *t* is fermentation time.

Concentrations of glucose, SA, and other organic acids were determined by HPLC (Waters, MA, USA) [8]. Unless specifically stated, all the concentrations of substrate and products in isFBB fermentation were calculated after subtracting the residues from the previous batches. The content of the free amino nitrogen (FAN) was measured using the ninhydrin reaction method as described in Lie’s study [49]. SA yield in this study refers to the amount of succinic acid (g) produced from the initial amount of glucose (g).

Apart from fermentations for metabolic evolution at any specific pH value (i.e., from 6.0 to 3.0 or even without control) were repeated more than three times until stable cell growth and SA production obtained, and the fed-batch fermentations were performed in duplicate; all other experiments were performed in triplicate. It should be pointed out that data in all tables of this study are shown as mean ± standard deviation.

## Additional file

**Additional file 1: Table S1.** Number of repeated batch fermentation, their corresponding pH and the names of evolved strains.

## Abbreviations

SA: succinic acid
isFBB: in-situ fibrous bed bioreactor
FAN: free amino nitrogen
PDC: pyruvate decarboxylase
ALDH: aldehyde dehydrogenase
DCW: dry cell weight
N.D: not detected
W.C: pH without control
TCA cycle: tricarboxylic acid cycle
