# Supplementary material for: Efficient metabolic evolution of engineered Yarrowia lipolytica for succinic acid production using a glucose-based medium in an in situ fibrous bioreactor under low-pH condition
Source: Biotechnol Biofuels. 2018 Aug 30;11:236. doi: 10.1186/s13068-018-1233-6 (PMC6116362; doi:10.1186/s13068-018-1233-6)
Supplement: Supplementary file 1 — Additional file 1: Table S1. Number of repeated batch fermentation, their corresponding pH and the names of evolved strains. [file 13068_2018_1233_MOESM1_ESM.docx]

Table S1. Number of repeated batch fermentation, their corresponding pH and the name of evolved strains.

| **Batch number** | **pH** | **Name of the strains** |
| --- | --- | --- |
| 1-8 | 6 | PSA02004 |
| 9-16 | 5 | PSA5.0 |
| 17-21 | 4 | PSA4.0 |
| 22-28 | 3 | PSA3.0 |
| 29-31 | Without control (i.e. 2.1-2.5) | PSA2.5 |
